# Assessment of Nutritional Status, Digestion and Absorption, and Quality of Life in Patients with Locally Advanced Pancreatic Cancer

**DOI:** 10.1155/2017/6193765

**Published:** 2017-08-20

**Authors:** J. E. Witvliet-van Nierop, C. M. Lochtenberg-Potjes, N. J. Wierdsma, H. J. Scheffer, G. Kazemier, K. Ottens-Oussoren, M. R. Meijerink, M. A. E. de van der Schueren

**Affiliations:** ^1^Department of Nutrition and Dietetics, Internal Medicine, VU University Medical Center, Amsterdam, Netherlands; ^2^Department of Radiology and Nuclear Medicine, VU University Medical Center, Amsterdam, Netherlands; ^3^Department of Surgery, VU University Medical Center, Amsterdam, Netherlands

## Abstract

**Background and Aim:**

To provide a comprehensive quantitative assessment of nutritional status, digestion and absorption, and quality of life (QoL) in patients with locally advanced pancreatic cancer (LAPC).

**Methods:**

Sixteen patients with LAPC were prospectively assessed for weight loss (WL), body mass index (BMI), fat-free mass index (FFMI), handgrip strength (HGS), dietary macronutrient intake, serum vitamin levels, resting and total energy expenditure (REE and TEE, indirect calorimetry), intestinal absorption capacity and fecal losses (bomb calorimetry), exocrine pancreatic function (fecal elastase-1 (FE1)), and gastrointestinal quality of life (GIQLI).

**Results:**

Two patients had a low BMI, 10 patients had WL > 10%/6 months, 8 patients had a FFMI < P10, and 8 patients had a HGS < P10. Measured REE was 33% higher (*P* = 0.002) than predicted REE. TEE was significantly higher than daily energy intake (*P* = 0.047). Malabsorption (<85%) of energy, fat, protein, and carbohydrates was observed in, respectively, 9, 8, 12, and 10 patients. FE1 levels were low (<200 *μ*g/g) in 13 patients. Total QoL scored 71% (ample satisfactory).

**Conclusion:**

Patients with LAPC have a severely impaired nutritional status, most likely as a result of an increased REE and malabsorption due to exocrine pancreatic insufficiency. The trial is registered with PANFIRE clinicaltrials.gov NCT01939665.

## 1. Introduction

Pancreatic cancer is the fourth most common cause of cancer death in Western countries with an overall 2-year survival rate of less than 10% [1–3]. Currently, surgical resection is the only curative option for patients with pancreatic cancer. However, approximately 80% of these patients present with unresectable locally advanced pancreatic cancer (LAPC) at the time of diagnosis, as a result of the late clinical presentation of disease symptoms [4]. The most common presenting symptoms are asthenia (86%), anorexia (85%), weight loss (85%), abdominal pain (79%), and choluria (59%) with jaundice [5, 6].

It is known that pancreatic cancer may affect the nutritional status through anorexia, an elevated resting energy expenditure (REE) as well as through exocrine pancreatic insufficiency (EPI), leading to fecal losses of energy, macro-, and micronutrients [7–11]. Subsequently, diarrhea, steatorrhea, and abdominal pain may affect quality of life (QoL) of patients [12].

Although pancreatic cancer has been associated with a poor nutritional status and QoL, little quantitative data of these aspects is available in patients with LAPC [9, 10, 13]. Most previous studies examined only one or two aspects of nutritional status or QoL in a group of pancreatic cancer patients, which has resulted in an incomplete overview of the contribution of each aspect to the decline of nutritional status in these patients. Therefore, the aim of this study is to describe a comprehensive assessment of the nutritional status, intestinal digestion and absorption, and QoL, including the contribution of exocrine pancreatic insufficiency in one group of patients with LAPC, to give a complete overview of the quantitative contribution of each aspect to the decline of nutritional status.

## 2. Patients and Methods

Sixteen outpatients with LAPC were prospectively included in the period between April 2014 and January 2015 at the interventional radiology ward of the VU University Medical Center (VUmc), Amsterdam, the Netherlands. A registered dietitian (JW) performed a comprehensive assessment of nutritional status (NS), digestion and absorption, and quality of life (QoL) within two weeks before experimental treatment with irreversible electroporation (IRE) of the pancreas [14].

The Medical Ethics Review Committee of the VUmc approved this study, and included patients gave written informed consent for participation before entering the study (PANFIRE—pilot study: irreversible electroporation (IRE) to treat locally advanced pancreatic carcinoma, registered at clinicaltrials.gov NCT01939665).

### 2.1. Patient Characteristics

Data of patient characteristics were obtained prospectively from medical charts, defined by sex, age at diagnosis, tumor location, previous tumor treatment, medication, use of pancreatic enzyme replacement therapy (PERT), and previous treatment.

### 2.2. Nutritional Status

The following parameters of nutritional status were determined: height, weight, body mass index, history of weight loss, body composition, handgrip strength, energy expenditure, and dietary energy and protein intake. Serum fat-soluble vitamin status was obtained from fasting blood samples.

#### 2.2.1. Height, Weight, Body Mass Index, and Body Composition

Height (cm) and weight (kg) were measured and performed with light clothing and without shoes. Body mass index (BMI, kg/m^2^) was calculated from weight in kilograms divided by height in meters squared. Weight loss history was inquired. Body composition was measured using bioelectrical impedance analyses using Bodystat© 1500MDD (EuroMedix) in patients without edema or ascites, in a supine position, after toilet visiting, and in a fasted state. Fat-free mass index (FFMI in kg/m^2^) was derived from fat-free mass in kilograms divided by height in squared meters.

The diagnostic criteria for malnutrition of The European Society of Clinical Nutrition and Metabolism (ESPEN) were used to define a poor nutritional status. These criteria include a BM < 18.5 kg/m^2^ or a combined finding of unintentional weight loss (>10% of habitual weight indefinite of time, or >5% over 3 months) and at least one of either reduced BMI (<20 or <22 kg/m^2^ in subjects younger and older than 70 years) or a low FFMI (<15 and <17 kg/m^2^ in females and males, resp.) [15].

#### 2.2.2. Handgrip Strength

Handgrip strength (HGS) was measured with a hydraulic analogue hand dynamometer (Baseline®) to obtain an indication of peripheral muscle function. Patients were instructed to perform three consecutive contractions with the nondominant hand, and the average power (kg) of the three consecutive measurements was used for analyses. Patients with a HSG below the 10th percentile (P10) of reference values of Bohannon et al. were considered to have a reduced peripheral muscle function [16].

#### 2.2.3. Energy Expenditure

Resting energy expenditure was predicted (REE) with the WHO'85 equation based on gender, age, and weight [17]. Resting energy expenditure (REE) was measured by using indirect calorimetry, based on oxide inhaling (VO_2_) and carbon dioxide exhaling (VCO_2_).

A ventilated hood system, metabolical monitor (Vmax Encore n29, Viasys), and Weir's equation were used to calculate REE. The measurements were performed in a supine position for 30 minutes in rest, without falling asleep, and patients were instructed to be fasted for at least 12 hours.

Patients with a measured REE (mREE) that deviated more than 10% from the predicted REE (pREE) by WHO'85 were considered to have a decreased or increased REE. Predicted total energy expenditure (TEE) was calculated by adding 30% activity factor to REE (resulting in pTEE; mTEE). To correct the REE for influence of the body composition, REE was divided by REE to calculate the REE in kcal/kg.

#### 2.2.4. Nutritional Intake

Patients were instructed to fill out a detailed 4-day food diary, accurately weighing all foods and drinks, using scales. Afterwards, all patients were interviewed by a registered dietitian (JW) to ensure all data was adequately documented. Average intake was calculated using a computerized food calculation program (based on The National Dutch Food Composition Table “NEVO 2006”). Total energy intake was determined by adding up the gross energetic value of fat (9.40 kcal/g), protein (4.40 kcal/g), and carbohydrates (4.10 kcal/g) [18]. Total energy intake was compared to energy requirements (mTEE and pTEE) to assess the energy balance. Protein intake was compared to protein requirements (≥1.2 g protein/kg/d) [19].

#### 2.2.5. Fat-Soluble Vitamin Status

Fasting samples of blood serum were taken at the medical laboratory of the VUmc.

The following vitamin levels were determined: vitamin A (retinol), D (25-OH), E (tocoferol), and the derivatives of vitamin K: international normalized ratio (INR) and activated partial thromboplastin time (APPT). Deficiencies were defined as the following: serum values of vitamin A < 1.2 *μ*mol/L, vitamin D < 50 nmol/L, and vitamin E < 20 *μ*mol/L. Reference values of international normalized ratio (INR) and activated partial thromboplastin time (APPT) are, respectively, 0.80–1.20 and 25–40 s. Patients with values above these references were suspected for a deficiency of vitamin K.

### 2.3. Digestion and Absorption

Assessment of digestion and absorption included analysis of fecal energy and nutrient losses and the fecal elastase-1 (FE1) test.

#### 2.3.1. Fecal Energy and Nutrient Losses

Patients were instructed to collect all 72-hour fecal specimens in a preweighted 5-liter bucket during days 2 to 4 of food intake recording. Patients using pancreatic enzyme replacement therapy (PERT) were instructed to stop using these enzymes at least two days before and during feces collection. All feces collected in 72 hours were weighed, homogenized, and stored at <4°C until analysis. Feces was analyzed for energy, fat, and nitrogen content.

A sample of feces was freeze dried until the process of bomb calorimetry, during which the sample was burnt, and the energy produced by the feces was measured as heat of combustion and calculated as fecal energy loss (kcal/d) [20, 21]. The process of bomb calorimetry was performed using a ballistic bomb calorimeter, (type CBB-33; Gallenkamp Manufactory, Etten-leur, The Netherlands) at the University of Groningen, The Netherlands.

Fecal fat content was determined using the method of van de Kamer et al. [22]. Fecal nitrogen content analysis was performed using the micro-Kjeldahl method [23]. Fecal protein content was calculated from the fecal nitrogen content by multiplying it by 6.25, assuming that all fecal nitrogen was derived from fecal protein. The amount of urinary protein losses was taken into account.

The carbohydrate content was calculated using the following equation (fecal energy − [fecal fat × 9.4] − [fecal protein × 4.4])/4.10. Finally, the intestinal absorption of energy was calculated as percentage of energy intake by ([total energy intake − fecal energy]/total energy intake) × 100. Intestinal absorption coefficients of fat, protein, and carbohydrates were calculated similarly [24, 25]. The procedure and reference values in healthy subjects of bomb calorimetry are extensively described by Wierdsma et al. [26]. In this study, an intestinal absorption capacity of <85% for either energy or fat, protein, or carbohydrates was defined as malabsorption. A fecal fat excretion of more than 20 g/d was defined as steatorrhea. This definition for steatorrhea was supposed to be clinically relevant to justify PERT.

#### 2.3.2. Fecal Elastase-1 Test

Exocrine pancreatic function was determined with the fecal elastase-1 (FE1) test.

FE1 was determined from a single feces sample. A FE1 below 200 *μ*g/g was defined as abnormal [27].

### 2.4. Quality of Life

QoL was objectified by the Gastrointestinal Quality of Life Index (GIQLI) questionnaire. This validated questionnaire assesses health-related quality of life of patients with gastrointestinal diseases [12].

Items of the GIQLI questionnaire were scored on a 5-point scale, in which the least desirable and most desirable answer scored, respectively, 0 and 4. The maximum total score was 144. The results were divided into four subscales: physical well-being, mental/physiological well-being, digestion, and defecation [12].

Scores of <55 were considered “unsatisfactory,” ≥55–70 “satisfactory,” ≥70–80 “ample satisfactory,” and ≥80 “very satisfactory.”

### 2.5. Statistical Analyses

Variables were described as mean and standard deviation (SD) if normally distributed and as median and interquartile ranges (IQR) if not normally distributed. Skewness and Kurtosis were used as indicators to test for normality of continuous variables. Independent *t*-tests and Wilcoxon (Mann–Whitney *U*) tests were performed to test for statistical differences between variables. A *P* value < 0.05 was considered to indicate statistical significance. Statistical analyses were performed in Statistical Package for Social Sciences (SPSS) version 22. The results of statistics that capture the performance of a diagnostic test are described as sensitivity (Se), specificity (Sp), and positive predictive value (PPV).

## 3. Results

### 3.1. Patient Characteristics

Information about patient characteristics is shown in Table 1.

### 3.2. Nutritional Status

The results of the assessment on nutritional status are shown in Table 2.

Based on the new ESPEN criteria for malnutrition, six patients were defined malnourished.

Fifty percent (8 patients) had a reduced peripheral muscle function based on a HGS below the 10th percentile.

Measured REE and TEE were significantly (*P* = 0.002) higher (33%; 457 kcal/d and 590 kcal/d, resp.) compared to predicted REE and TEE. Median REE per kg FFM was 34.7 (33.3–49.9) kcal/kg. mTEE was significantly higher (mean difference 423 kcal, IQR −289; +776 kcal) than mean daily energy intake (*P* = 0.047), but pTEE was not. Mean protein intake was 1.0 g/kg, which is lower than the predefined requirement of ≥1.2 g protein/kg/d, not yet corrected for fecal loss of proteins.

Vitamin A and E deficiencies were present in 2/16 patients, and vitamin D (25-OH) deficiency in 9/16 patients; none of the patients had used vitamin A, D, or E supplements. None of the patients had an INR or APPT above the reference values.

### 3.3. Digestion and Absorption

Table 3 shows the results of the assessment of digestion and absorption.

Thirteen out of 15 patients had malabsorption for one or more macronutrients. Out of the five patients that had been prescribed PERT, three had a completely normal fat absorption (>85%) and normal fecal fat excretion (<20 g/d). Besides, 5/15 patients who demonstrated fat malabsorption and increased fecal fat excretion had not been prescribed PERT (data not shown). Median (IQR) FE1 was 61 (21.5–147.5) *μ*g/g, whereas thirteen patients showed a FE1 < 200 *μ*g/g.

FE1 had a PPV of 0.54, a Se of 1.0, and a Sp of 0.22 for fecal fat excretion/steatorrhea (>20 g/d). Seven out of 13 patients with a decreased FE1 had no steatorrhea and a normal fat absorption capacity > 85% (6/13).

### 3.4. Quality of Life

Twelve patients completed the GIQLI (items of the subscale mental well-being were missing in 2 patients; the GIQLI of one patient was not returned). “Very satisfactory” was the most observed score (83%) of QoL. QoL was never assessed as unsatisfactory, once as satisfactory, and once as ample satisfactory. Scores of the total assessment of QoL and scores for each subscale are shown in Table 4.

## 4. Discussion and Conclusion

This study shows that patients with LAPC generally have a severely impaired nutritional status, most likely as a result of an increased REE and malabsorption (increased fecal losses of energy macronutrients and micronutrients) due to exocrine pancreatic insufficiency.

These results provide an in-depth picture of problems of nutritional status, digestion and absorption capacity, and QoL in patients with LAPC.

### 4.1. Nutritional Status

Although most of the patients had lost a considerable amount of preillness weight in the preceding 6 months, our patients presented mostly with a normal BMI. In contrast, FFMI and peripheral muscle function were low in half (50%) of the patients.

In this study, measured REE was significantly higher (33%) than predicted requirements (by WHO'85 equation). This is in line with a previous study which demonstrated an increased REE (33%) in patients with pancreatic cancer compared with healthy controls, suggesting hypermetabolism in patients with advanced pancreatic cancer [9]. However, another more recent study found no difference between REE of pancreatic cancer patients and healthy controls after correcting for lean body mass [28]. As equations underestimated energy requirements in our study population, our advice would be to use equations only if measuring REE is not practically achievable. Adding another 30% for hypermetabolism may then be a pragmatic approach.

According to the estimated energy requirements, energy intake was apparently sufficient. However, according to measured energy requirements, the energy intake was deficient by approximately 423 kcal/d. The median daily protein intake of 1.0 g/kg was generally below the predefined requirements of 1.2 g/kg [19]. However, this deficiency is even worse since the median protein intake has to be corrected for fecal protein losses. After correction, this result in a net median protein availability of 0.72 (0.34–0.87) g/kg/d, which is far below predefined requirements.

The prevalence of vitamin A and E deficiency was 13% (2/16), most likely due to fat malabsorption in at least half of the patients. However, since the physical storage of fat-soluble vitamins is enough to prevent deficiencies for months to years, sufficient levels of fat-soluble vitamins may be expected [29]. Fifty-six percent (*N* = 9) of the patients in this small study group suffered from a vitamin D 25-OH deficiency (according to the Dutch cut-off point for a vitamin D value below 50 *μ*mol/L), which is slightly higher than the prevalence of 50% in the “independently living elderly” in The Netherlands [30]. Since patients with LAPC might be at increased risk of developing deficiencies of fat-soluble vitamins over time, due to ongoing fecal losses, it is recommended to monitor the status of fat-soluble vitamins. If deemed needed, additional supplementation should be given.

### 4.2. Digestion and Absorption

Malabsorption of energy and macronutrients is an important problem in patients with LAPC. This study showed substantial individual differences in intestinal (energy) absorption capacity, which could not be explained by location of the tumor.

Most likely, decreased intestinal absorption capacity was the result of an exocrine pancreatic dysfunction since 13/15 patients had a decreased level of FE1. FE1 is a specific human protease synthesized by the acinar cells of the pancreas. It is stable during transit, and its detection in the stool is uncomplicated [27]. However, in patients with intestinal failure, a short intestinal transit time may result in low FE1 levels as well. In our study population, no symptoms of a short intestinal transit time were found.

Interestingly, 5 patients had been prescribed PERT. This proved to be incorrect in 4 (80%), who displayed a normal fat excretion (<20 g/d) and intestinal absorption capacity for fat (>85%). In contrast, 6 patients had not been prescribed PERT, while demonstrating steatorrhea. Furthermore, FE1 as a marker for exocrine pancreatic insufficiency failed to detect steatorrhea as it appeared false positive in 7 out of 15 patients. This suggests that there is room for improvement of prescription of PERT. Based on our study results, PERT should be better prescribed on individual quantitative fecal fat measurements, optionally in combination with FE1, rather than on either clinical presentation, FE1 level, or as a standard procedure.

### 4.3. Quality of Life

Of the four subscales for QoL, physical and mental well-being scored lower than digestion and absorption. This could be related to a poor nutritional status, low HGS, and low FFMI which may affect functional status and thereby independence and self-reliance of patients. Still, overall quality of life was rated satisfactory or higher in almost all patients. This suggests that patients adapt to their clinical situation and adjust their QoL expectancies. The higher scores for digestion and defecation in this study could be explained by frequently contact with the dietitian with attention for EPI and fecal losses, leading to decrease of clinical symptoms of malabsorption.

### 4.4. Strengths and Weaknesses

To our knowledge, this is the first study which extensively assessed nutritional status, digestion, intestinal absorption capacity, and quality of life, combining a broad scala of nutritional assessment techniques in one group of patients with LAPC. A limitation is the relatively small sample size (which was directly linked to the maximum eligible number of patients in the IRE study) and the cross-sectional design. Therefore, the results should be interpreted with caution and no causal links can be drawn.

Future research is recommended to assess the nutritional status, digestion and absorption, and QoL prospectively after experimental treatment with IRE.

## 5. Conclusions

In conclusion, patients with LAPC are at high risk for developing a poor nutritional status and poor physical functioning, most likely due to a combination of increased energy expenditure and malabsorption. Optimizing pancreatic enzyme supplementation in combination with hyperalimentation to correct energy and protein intake for fecal losses may be a strategy to counteract these symptoms as long as patients are actively treated. An individual approach seems necessary given the large differences between patients.

## Figures and Tables

**Table 1:** Characteristics of the group of patients with LAPC at baseline.

Patients	*N* = 16
Male/female, *n*	8/8
Mean age (years) (SD)	60.3 (9.5)
Pancreas tumor location	
Head	10
Tail	1
Uncinate process	5
Previous treatment (yes/no)^1^	14/2
Patients with pancreatic enzyme replacement therapy^2^	5
Dietetic treatment (yes/no)^3^	10/6

^1^Previous treatment being: chemotherapy (CT) (*N* = 3), percutaneous transhepatic cholangiography biliary drainage (PTC-drain) or plastic endoprothese (*N* = 3), CT and PTC-drain (*N* = 2), gastrojejunostomy (GJ) (*N* = 1), hepaticojejunostomy (HJ) (*N* = 2), CT and HJ (*N* = 1), GJ and HJ (*N* = 1), and CT, GJ, and HJ (*N* = 1). ^2^Patients commenced enzyme treatment varying from 2 months till 3 weeks before the assessment. ^3^Patients received dietetic treatment from a dietitian varying from 2 months till 10 days before the assessment.

**Table 2:** Results of the parameters of the nutritional assessment.

Parameter	Mean (SD) or median (IQR)^1^	Criteria or reference value	Number of patients
Height (cm)	172 (9)		
Weight (kg)	70.3 (8.4)		
BMI (kg/m^2^)	23.9 (2.5)	BMI < 18.5 kg/m^2^	*N* = 0
		BMI < 20 (<70 y) or <22 (≥70 y) kg/m^2^	*N* = 2
Weight loss in past 6 months (%)^2^	13 (6–17)	Weight loss > 10% in 6 months	*N* = 10
Fat-free mass (kg)	M: 54.0 (49.8–58.0)		
	F: 41.0 (38.3–43.8)		
Fat-free mass index (kg/m^2^)	M: 17.0 (1.7)	FFMI < 17.0 (M) or <15.0 (F) kg/m^2^	*N* = 8
	F: 14.9 (0.9)		
Handgrip strength (kg)	M: 33.7 (8)	HGS < P10	*N* = 8
	F: 24.8 (6)		
REE (kcal/d)^3^	m 1829 (1622–2030)		
	p 1372 (1316–1440)		
mREE/pREE (%)^3^	133 (115–147)		
TEE (kcal/d)^3^	m 2378 (2109–2639)		
	p 1786 (1730–1885)		
	m-p = ∆590 (276–736)		
Energy intake (kcal/d)^4^	1926 (1681–2283)		
Protein intake (g/kg/d)^4^	1.0 (0.4)	<1.2 g/kg/d	*N* = 10
Vitamin A (*μ*mol/L)	2.1 (1.3–2.5)	<1.2 *μ*mol/L	*N* = 2
Vitamin E (*μ*mol/L)	25 (22–31)	<20 *μ*mol/L	*N* = 2
Vitamin D (25-OH) (nmol/L)	46 (34–70)	<50 nmol/L	*N* = 9
APPT (s)	34 (32–37)	>40 s	*N* = 0
INR (s)	1.03 (0.98–1.05)	>1.10 s	*N* = 0

^1^Mean (SD) in case of normally distributed variables and median (IQR) in case of not normally distributed variables. ^2^*N* = 14: two persons could not recall their historical weight (pregnancy and unknown reason). ^3^*N* = 15: one patient skipped the REE measurement because of too much mental stress/tiredness during the test day. ^4^*N* = 15: one patient could not start a food diary because of an emergency IRE treatment. M: male; F: female; m: measured; p: predicted by WHO'85; REE: resting energy expenditure; TEE: total energy expenditure.

**Table 3:** Fecal energy losses, intestinal absorption capacity, and exocrine pancreatic function.

	Average daily intake	Fecal losses median [IQR]	Absorption capacity (%) median [IQR]	Absorption capacity < 85%
Weight (g/d)	—	236.5 (97.8–409.3)	—	—
Energy (kcal/d)	1926 (1681–2283)	324 (198–597)^1^	82.9 (47.8–87.9)	*N* = 9
Fat (g/d)	86.0 (62.0–105.0)	8.7 (3.8–29.9)	84.2 (39.1–93.0)	*N* = 8
Nitrogen (g/d)	—	2.6 (1.5–3.4)	—	—
Protein (g/d)	74.0 (62.8–88.3)	16.3 (9.4–21.3)	77.0 (54.5–81.6)	*N* = 12
Carbohydrates (g/d)	213.0 (135.0–246.0)	39.6 (24.8–54.5)	80.6 (62.7–85.0)	*N* = 10

*N* = 15: one patient could not collect feces because of logistical problems.^1^Reference of healthy subjects is 200 kcal/d [22]. Loss of >200 kcal/d, *N* = 12.

**Table 4:** Results of the QoL assessment by GIQLI in 13 patients with LAPC.

GIQLI	Total item score median (IQR)	Score range	Mean sum as % of maximum score	Mean item score median (IQR)	Number of items in the GIQLI
Total^1^	102 (82.5–118.5)	0–144	71	2.8 (2.3–3.3)	36
Physical well-being^2^	25.5 (16.8–30.0)	0–40	65	2.6 (1.7–3.0)	10
Gastrointestinal digestion^2^	31 (20–34)	0–40	78	3.1 (2.0–3.4)	10
Gastrointestinal defecation^2^	20 (17.5–22)	0–24	83	3.3 (2.9–3.7)	6
Mental well-being^1^	12 (10–13.3)	0–20	60	2.40 (2.00–2.65)	5

^1^ *N* = 12: one patient did not return the questionnaire and 3 patients did return incomplete questionnaires for subscale mental well-being. ^2^*N* = 14: one patient did not return the questionnaire and 1 patient returned an incomplete questionnaire for subscales physical well-being, gastrointestinal digestion and gastrointestinal defecation.

## Abbreviations

BMI: Body mass index
EPI: Exocrine pancreatic insufficiency
FE1: Fecal elastase-1
FFMI: Fat-free mass index
GIQLI: Gastrointestinal quality of life index
HGS: Handgrip strength
IRE: Irreversible electroporation
JW: J. Witvliet-van Nierop (author and dietitian)
LAPC: Locally advanced pancreatic cancer
m: Measured
NS: Nutritional status
p: Predicted
PERT: Pancreatic enzyme replacement therapy
QoL: Quality of life
REE: Resting energy expenditure
TEE: Total energy expenditure
VUmc: VU University Medical Center
WHO'85: Equation of World Health Organization 1985 to predict REE
INR: International normalized ratio
APPT: Activated partial thromboplastin time.
